# Managing emerging transnational public health security threats: lessons learned from the 2014 West African Ebola outbreak

**DOI:** 10.1186/s12992-018-0396-z

**Published:** 2018-07-27

**Authors:** Aaron M. Wendelboe, Micah McCumber, Julie Erb-Alvarez, Nicholas Mould, Richard W. Childs, James L. Regens

**Affiliations:** 1 Department of Biostatistics and Epidemiology, University of Oklahoma Health Sciences Center, 801 NE 13th St., CHB 323, Oklahoma City, OK 73104 USA; 2 Center for Intelligence and National Security, University of Oklahoma, 755 Research parkway, Suite 520, Oklahoma City, OK 73104 USA; 3 Indian Health Service, US Public Health Service, 701 Market Drive, Oklahoma City, OK 73114 USA; 4 National Heart Lung and Blood Institute, Hematology Branch, Section of Transplantation Immunotherapy, National Institutes of Health, Building 10-CRC, Room 3-5330, Bethesda, MD 20814 USA; 5 Division of Intramural Research, Office of the Clinical Director, National Institutes of Health, Bethesda, MD USA

**Keywords:** Pandemic, Public health, Response, Outbreak investigation, Ebola virus disease

## Abstract

**Background:**

Pandemics pose significant security/stability risks to nations with fragile infrastructures. We evaluated characteristics of the 2014 West African Ebola outbreak to elucidate lessons learned for managing transnational public health security threats.

**Methods:**

We used publically available data to compare demographic and outbreak-specific data for Guinea, Sierra Leone, and Liberia, including key indicator data by the World Health Organization. Pearson correlation statistics were calculated to compare country-level infrastructure characteristics with outbreak size and duration.

**Results:**

Hospital bed density was inversely correlated with longer EVD outbreak duration (*r* = − 0.99). Country-specific funding amount allocations were more likely associated with number of incident cases than the population at-risk or infrastructure needs. Key indicators demonstrating challenges for Guinea included: number of unsafe burials, percent of EVD-positive samples, and days between symptom onset and case hospitalization. Sierra Leone’s primary key indicator was the number of districts with ≥1 security incident. Liberia controlled their outbreak before much of the key-indicator data were collected.

**Conclusion:**

Many of the country-level factors, particularly the WHO key indicators were associated with controlling the epidemic. The infrastructure of countries affected by communicable diseases should be assessed by international political and public health leaders.

## Background

The nexus between risks posed by infectious disease pandemics to the stability of fragile states and international security was demonstrated dramatically by the largest reported Ebola Virus Disease (EVD) outbreak which occurred in West Africa between 2014 and 2015 [1–5]. Underscoring its severity, the 2014 West African Ebola outbreak was larger than all previous EVD outbreaks combined. The epidemic began in December 2013 and the World Health Organization (WHO) was notified on March 23, 2014. The WHO declared it a public health emergency of international concern on August 8, 2014 [6]. Further demonstrating the security threat posed by this public health emergency, the United Nations Security Council Resolution 2176 (2014) adopted on September 15, 2014 declared the outbreak was “a threat to international peace and security in the region” [7]. The international community’s response acting in cooperation with the immediately affected countries included diagnosing the disease as early as possible, contact tracing, patient isolation and care, infection control, ring vaccination, safe burial practices, and public education (e.g., messages on billboards and radio describing symptoms, hand hygiene, school closures, and avoiding close contact to prevent transmission). Indeed, many of these factors comprise the majority of the WHO Ebola Situation Reports’ key performance indicators [8]. The outbreak officially ended on March 29, 2016. Ten countries were directly impacted, three of which experienced significant outbreaks (Guinea, Liberia, and Sierra Leone), while seven countries reported one or more EVD cases without widespread human-to-human transmission (Italy, Mali, Nigeria, Senegal, Spain, the UK, and the US) [9, 10].

We aim to systematically examine country-specific factors within the context of the larger global pandemic – with the greatest emphasis on Guinea, Liberia, and Sierra Leone. Specifically, we 1) conduct a quantitative analysis of country-specific factors in Guinea, Liberia, and Sierra Leone and 2) conduct a qualitative analysis of patterns of disease incidence and transmission among all countries with ≥1 case of EVD to draw lessons learned from the 2014 West African Ebola outbreak for managing emerging transnational health security threats.

## Results

### Overall summary

**Fig. 1:**
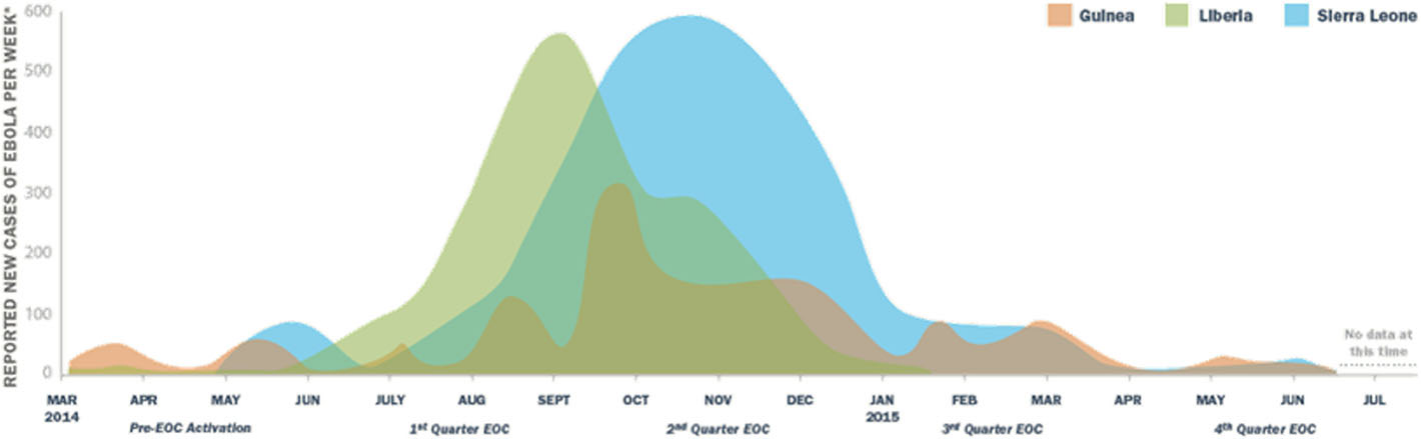
Epidemic Curve of Ebola Cases in West Africa. Reprinted with permission from CDC, cdc.gov/about/ebola/timeline.html

By March 30, 2016, WHO reported 28,646 cases and 11,323 deaths from the 2014 West African Ebola Outbreak, [9] with an overall case fatality rate of 39·5%. Cases were observed in 10 countries across three continents (Africa, Europe, and North America). Table 1 summarizes the number of cases, deaths, contacts followed, and date the country was first declared Ebola-free. Epidemic curves for the three widely affected countries are shown in Fig. 1. At the epidemic’s peak, the doubling time was 15–20 days for cases in Liberia and 30–40 days for Sierra Leone and Guinea [11]. Although four new confirmed cases were diagnosed in Guinea during March 17–28, the WHO Director-General declared on March 29, 2016 the end of the Public Health Emergency of International Concern regarding the EVD outbreak in West Africa [9].

**Table 1:** Distribution of Cases, Deaths, Contacts, and Date declared Ebola-free

Country	Cases	Deaths	Case-fatality Rate (%)	HCW Cases	HCW Deaths	Contacts	Date first Ebola-Free^a^
Widely affected							
Guinea	3811	2543	66.7	187	94	TMTC	December 29, 2015
Sierra Leone	14,124	3956	28.0	303	221	TMTC	November 7, 2015
Liberia	10,675	4809	45.0	378	192	TMTC	May 9, 2015
Limited transmission							
Italy	1	0	0.0	1	0	19 [18]	July 20, 2015
Mali	8	6	75.0	2	2	433 [38]	January 18, 2015
Nigeria	20	8	40.0	0	0	891 [22]	October 20, 2014
Senegal	1	0	0.0	0	0	74 [39]	October 17, 2014
Spain	1	0	0.0	1	0	83 [17]	December 2, 2014
United Kingdom	1	0	0.0	1	0	55 [8] (+ 177 airplane)	March 10, 2015
United States	4	1	25.0	3	0	177 [40]	December 20, 2014

^a^Each of the widely-affected countries was declared Ebola-free and subsequently had a small number of newly detected cases. The first date of being declared Ebola-free is a reasonable marker of when the outbreak was controlled in that country

HCW – Healthcare worker

TMTC – Too many to count

### Outbreak by widely-affected country

#### Guinea

**Fig. 2:**
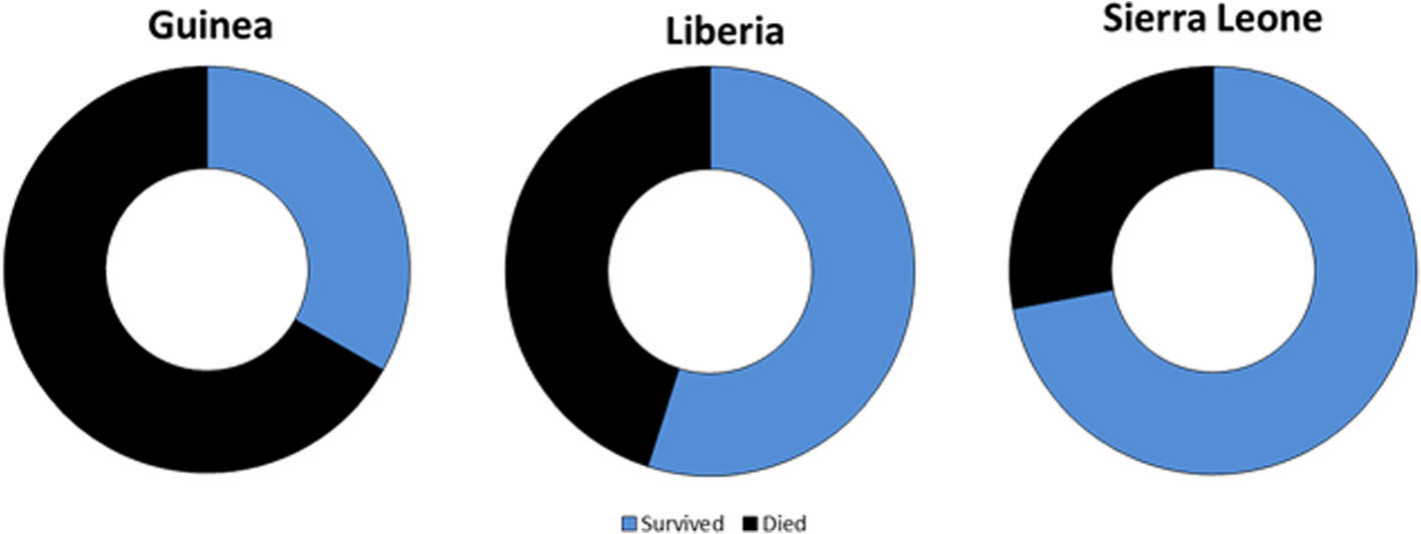
Case fatality among affected countries as of March 30, 2016

The first case likely occurred December 26, 2013, but not identified as Ebola until March 21, 2014 [12]. The index case was traced to a two-year-old boy in Meliandou, a village near Gueckedou, Guinea [13]. Evidence suggests his exposure was likely an infected Angolan free-tailed bat [13, 14]. Although Guinea had the highest case fatality rate (66·7%, Fig. 2) and was the last to be declared Ebola-free, it had the lowest number of cases among the widely-affected countries. The peak number of weekly cases reported was 526 in November 2014 [15]. Eight (23·5%) of the 34 prefectures in Guinea did not report any Ebola cases [16]. By March 29, 2016, there were 3811 EVD cases, 2543 deaths [17] (Table 1) and 187 cases among health care workers (HCWs), of which 94 died [18]

.

#### Sierra Leone

The first Sierra Leone case was retrospectively identified as a female guest in the index case’s home in Meliandou, Guinea, but returned to Sierra Leone and died in early January 2014 [12]. It was not until April 1, 2014 that Sierra Leone “stepped up vigilance for imported cases when two members of the same family who had died from Ebola virus disease in Guinea were repatriated to Sierra Leone for burial” [12]. By November 2014, EVD had been reported in each district in Sierra Leone [16] and the peak number of weekly cases reported was 1997 [15]. Sierra Leone had the highest number of cases (14,124) but the lowest case fatality rate (28·0%). The cumulative number of deaths was 3956 [8] (Table 1) and 303 EVD cases among HCW, of which 221 died [18].

#### Liberia

Ebola was first reported on March 30, 2014 in Lofa’s Foya district, [12] which borders Sierra Leone and Guinea. Lofa became the first model area of success when it reported no new cases October–December 2014. Success was attributed to “implementing the full package of control interventions, including community engagement, acceptance, and ownership of the response” [12]. Ultimately there were 10,675 cases and 4809 deaths (case fatality rate = 45·0%) [8]. All districts in the country reported cases of EVD [16]. Liberia had the highest number of HCW EVD cases (*n* = 378), but fewer deaths (*n* = 192) [18] than Sierra Leone.

### Comparison of widely-affected countries

Table 2 illustrates important demographic differences between these three countries. Guinea’s population (11,474,383) is approximately twice Sierra Leone’s (5,743,725) and Liberia’s (4,092,310), yet has the lowest health and education expenditures and literacy rate. Guinea also has the lowest ethnic diversity.

**Table 2:** Population Statistics for Guinea, Sierra Leone, and Liberia [41]

Characteristic	Guinea	Sierra Leone	Liberia
Population	11,474,383	5,743,725	4,092,310
Population density per km^2^	40.9	79.4	40.4
Median age (years)	18·7	19	17·9
Infant mortality rate (deaths/1000 live births)	55·24	73·3	69·19
Health expenditures (% of GDP)	6	18·8	19·5
Physician density (physicians/1000 population)	0·1	0·02	0·01
Hospital bed density (beds/1000 population)	0·3	0·4	0·8
Literacy (% age 15+ can read and write)	41	43·3	60·8
Education expenditures (% of GDP)	2·5	2·9	2·8
Urbanization (% urban)	35·4	39·2	48·2
Religions	Muslim 85%, Christian 8%, Indigenous beliefs 7%	Muslim 60%, Christian 10%, Indigenous beliefs 30%	Christian 85·6%, Muslim 12·2% Traditional 0·6% Other 0·2% None 1·4%
Ethnic groups	Peuhl 40%, Malinke 30%, Soussou 20%, other 10%	Temne 35%, Mende 31%, Limba 8%, Kono 5%, Kriole 2%, Mandingo 2%, Loko 2%, Other 15%	Kpelle 20·3%, Bassa 13·4%, Grebo 10%, Gio 8%, Mano 7·9%, Kru 6%, Lorma 5·1%, Kissi 4·8%, Gola 4·4%, other 20·1%

Generally, we failed to detect correlations between the health indicator data (infant mortality rate, health expenditures as % of GDP, physician density, and hospital bed density) and EVD-related outcomes (i.e., duration of the EVD outbreak and the EVD-specific mortality rate). The only statistically significant correlation was between outbreak duration and hospital bed density (*r* = − 0·99, *p* < 0·01); fewer hospital beds per 1000 population was correlated with longer EVD outbreak duration.

Country-specific information regarding the distribution of EVD-related financial resources is opaque. Initially, Guinea received approximately half the funding Sierra Leone and Liberia received. According to a WHO budget [19] for July–December 2014, Guinea received $1,927,993, Sierra Leone received $4,471,599, and Liberia received $3,711,908. Nigeria, which ultimately was not widely affected, initially received nearly as much funding ($1,305,000) as Guinea. A WHO financial report covering October 31, 2015 reported the following geographic allocation of donor funds: Guinea 11%, Liberia 32%, Sierra Leone 22%, Regional 33%, and Other 2% [20]. By the outbreak’s end, approximately $3·7 billion had been dedicated to the Ebola response during 2014–2016 [21]. Money allocation more closely correlated with country-specific incident EVD cases than population size. The EVD case and population-at-risk distribution was: Guinea = 13·3% cases, 53·8% population-at-risk, Sierra Leone = 49·3% cases, 27·0% population-at-risk, and Liberia = 37·3% cases, 19·2% population-at-risk.

**Fig. 3:**
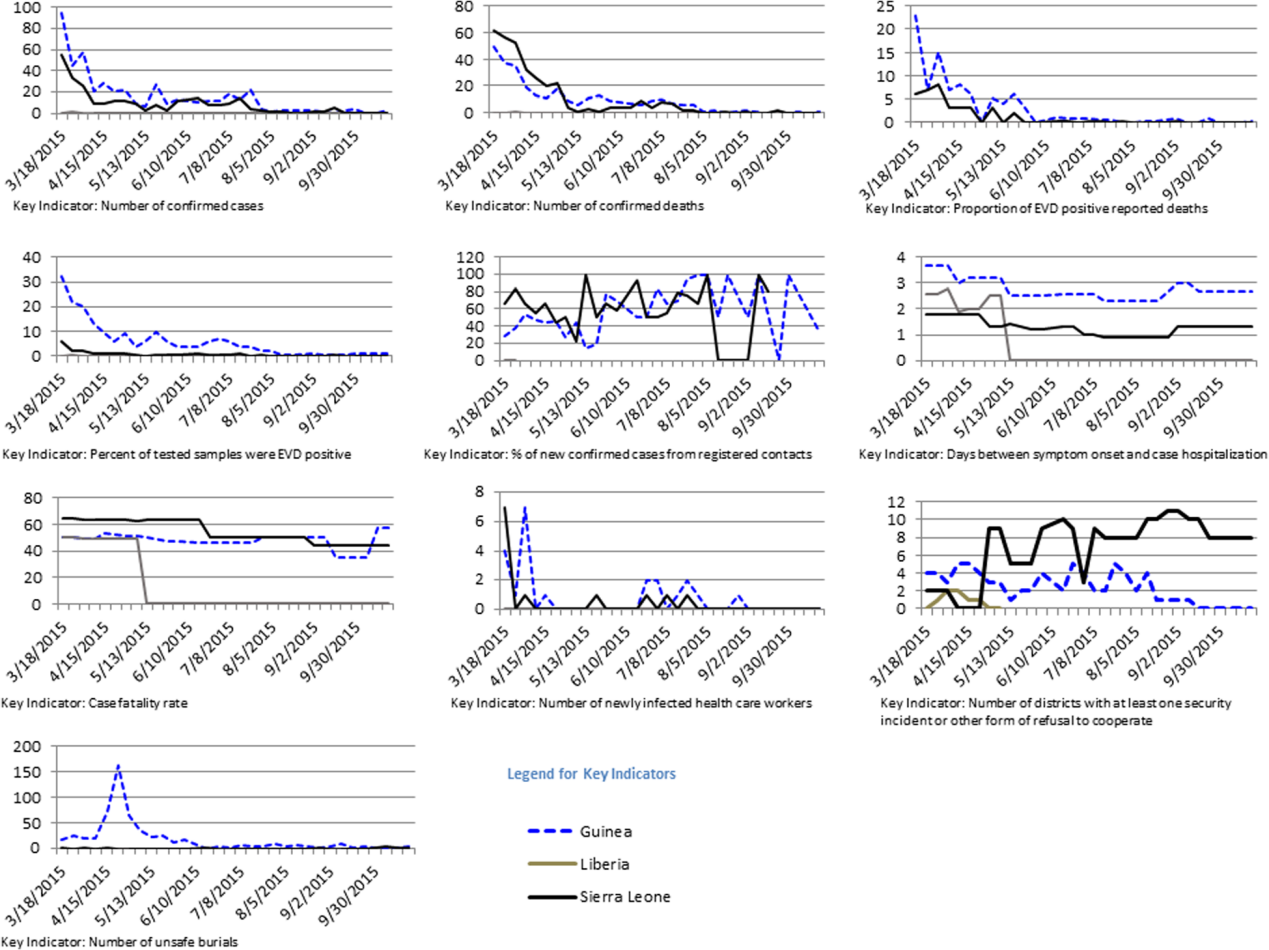
Indicator data from WHO Ebola Situation Report

Comparing the 10 key indicator data provides limited insights into differences between Guinea and Sierra Leone’s country-specific epidemics; the epidemic was largely controlled in Liberia (Fig. 3) by the time they were systematically collected. Three indicators showed worse problems in Guinea (number of unsafe burials, percent of tested samples that were EVD positive, and days between symptom onset and case hospitalization) and one showed worse problems in Sierra Leone (number of districts with at least one security incident); the remaining indicators were nearly equivalent (Fig. 3).

### Outbreak by country with limited transmission

EVD cases were exported to seven additional countries and details regarding the country-specific outbreaks are organized by date of first confirmed EVD case. Of these, Nigeria, Senegal, and Mali are West African countries with relatively high potential for ongoing transmission, particularly Nigeria. However, a robust public health response prevented widespread transmission in these countries [12]. The US, Spain, the UK, and Italy also had ≥1 imported EVD case. Only the US reported in-country EVD transmission. All of these countries reported their EVD cases in 2014, except Italy, which was in May 2015. Table 1 summarizes the number of cases, deaths, contacts followed, and date first declared Ebola-free.

#### Nigeria

The first case was reported July 20, 2014 in the Lagos District and imported from Liberia [12]. This marks the first recorded time Ebola spread to another country via airline travel.[12]Although the index case in Nigeria transmitted the disease to at least five people who died, no transmission occurred on the flight, despite the infected adult male vomiting enroute [12]. Port Harcourt was Nigeria’s second district to be affected with an EVD case reported on August 1. Nigeria was declared Ebola-free on October 20, 2014 with a final case count of 20, eight deaths [8] and ≥ 891 contacts requiring follow-up [22].

#### Senegal

An adult male who travelled by road from his home in Guinea to Dakar was Senegal’s only EVD case and reported on August 29, 2014 [12]. He survived and Senegal was declared Ebola-free on October 17, 2014.

#### United States of America

On September 30, 2014, the first EVD case in the US was reported in Dallas, TX. An adult male travelling from Liberia developed symptoms four days after arriving and expired on October 8. He transmitted EVD to two female nurses. The first was diagnosed on October 10 and discharged from the hospital on October 24. The second was diagnosed on October 15 and discharged on October 28. She took two flights but no transmission was identified [23]. The fourth case was in a male physician living in New York City who worked with Doctors without Borders. He was diagnosed October 24 and discharged November 11. This physician potentially exposed multiple individuals during the first day of his illness at public places like restaurants and a bowling alley, but no transmission was identified [23]. Of these four cases, one died, two were imported, and two were from local transmission [8]. There were an additional six EVD cases acquired in West Africa but treated in the US [24]. Of these, there were three HCWs, an aid worker, a missionary, and a cameraman. All but one recovered. The U.S. was declared Ebola-free on December 20, 2014.

#### Spain

An adult male, Spanish HCW was repatriated from Sierra Leone on September 22, 2014 and reported as an EVD case on October 6 [25]. Successful contact tracing of 87 people was conducted and no subsequent transmission was identified. He survived and Spain was declared Ebola-free on December 2, 2014.

#### Mali

Health officials in Mali investigated six suspect cases in April 2014 which helped prepare for the introduction of their first confirmed case in October 2014. This EVD case was a two-year-old female from Guinea (reported on October 23) [12]. Being symptomatic during her journey to Mali, the girl died the following day. On October 25, a Grand Imam from Siguiri prefecture in Guinea was admitted to a clinic in Bamako for acute kidney failure. Ebola was not initially recognized and he died on October 27. Because his symptoms began in Guinea, he is reported as a Guinean case. That case led to seven additional cases, with five deaths [12]. By January 18, 2015, when Mali was declared Ebola-free, the final case count was eight with six deaths [8]. Kayes and Bamako were the only districts affected [16].

#### United Kingdom

On December 29, 2014 a female HCW returning from Sierra Leone was reported as the only EVD case in the UK [26]. She developed symptoms as she arrived in London. The 177 people on a shared flight were classified by at least one source as exposed contacts, but were treated as low risk. All 132 passengers on board the shared flight (AT0800 from Casablanca to London) and crew members were successfully contacted. Public Health England advised passengers sitting in the two rows adjacent (comprising 21 passengers) to take their temperature twice daily for 21 days [27]. The patient survived and there were no additional cases [16].

#### Italy

On May 12, 2015, a case of Ebola in a male Italian volunteer HCW was reported. He returned from Sierra Leone on May 7, became symptomatic on May 10, and hospitalized on May 11. No inflight contact tracing was done. There were 19 contacts to this case [18]. On July 20, 2015, Italy was declared Ebola-free having no additional EVD cases and no deaths [28].

## Discussion

WHO declared the Ebola epidemic over on March 29, 2016 with 28,646 cases and 11,323 deaths across 10 countries and three continents. Of the three countries most widely affected, Liberia was first to successfully control the epidemic (February 2015), which was before the key indicator data were collected (March 2015). The epidemic in Sierra Leone and Guinea was largely controlled by April 2015, but experienced sustained transmission into November 2015.

Using the data from the present study, the key indicators that seemed to be substantially different between Sierra Leone and Guinea were 1) the number of unsafe burials, 2) the percent of tested samples that were EVD positive, 3) the number of days between symptom onset and case hospitalization, and 4) the number of districts with at least one security incident. There are a number of additional factors highlighted in the WHO Ebola Situation Reports (and substantiated in the published literature). Burial practices were cited as the single strongest risk factor for transmission; 60–80% of all cases reported an exposure to a traditional burial [29]. While previous outbreaks were largely controlled by using contract tracing, [10, 30] this is the first EVD pandemic with a prolonged duration where treatment and supportive care played an unquantified role in the response [10, 30, 31]. Another key difference was the strong stigma associated with EVD infection in this outbreak as demonstrated by community attempts to hide cases from authorities. These areas came to be known as “shadow zones” [22] and were accompanied by angry mobs attacking health care facilities and workers. The first such occurrence was on April 4, 2014 in Macenta, Guinea, [27] and differences in events between Sierra Leone and Guinea are shown in Fig. 3.

The Pearson correlation estimates were limited by small sample size (three countries). The strongest country-level health indicator was between hospital bed density and the duration of the outbreak (*r* = − 0·99). Increasing bed capacity was shown to contribute to controlling the Ebola pandemic by two modeling studies, [32, 33] however, the belated timing of the escalation of bed capacity likely limited its impact [34]. We suggest that helping countries increase health infrastructure, particularly by having more hospital beds, as well as ongoing infection control training and capacity building, may help combat severe diseases such as EVD. Diagnostic equipment, largely unavailable during the early part of the outbreak, was also key for effective patient management.

This pandemic highlights the need for the international community to respond to transnational outbreaks in a timely manner. Despite the heroic efforts of early responders (e.g., local HCWs, personnel with *Médecins Sans Frontières, CDC, and others), on a global scale,* the response time was inadequately slow. This is possibly due to international politics and the sluggish turning wheels of governments who strive to do the right thing while meeting the needs of their citizens.

The response by the US arguably began in March 2014 when CDC deployed personnel to investigate Ebola cases in Guinea, and further on July 9, 2014 with the activation of CDC’s Emergency Operations Center. However, because EVD was largely out of the public’s eye until the fall of 2014, its response was similarly delayed. That is, the peak number of EVD cases in Liberia was September 21, 2014; one week after President Obama’s announcement to commit 3000 troops and provide additional aid to the Ebola response effort [35] and one month after WHO declared it a public health emergency of international concern. These events unlikely contributed substantially to controlling the EVD pandemic [34]. The US’s response was impressive in terms of funding and personnel. By April 2015, the US spent $1.4 billion on the Ebola response effort. During 2014–2016, the HHS global and domestic response mobilized more than 4000 personnel [36]. Approximately 300 active duty Commissioned Corps officers in the US Public Health Service established, operated and staffed the Monrovia Medical Unit, an Ebola Treatment Unit having more sophisticated medical capabilities than those in conventional Ebola treatment Units. The Monrovia Medical Unit provided advanced supportive care to responders who became ill during this West African crisis and were the only US (non-private) personnel to provide direct patient care to persons infected with EVD.

This study is subject to certain limitations. Perhaps the most substantial of which is the difficulty to quantify many factors affecting control of the outbreak. For example, the West African people educated their communities, facilitating the change of social practices, such as greetings with physical contact and modifying long-standing traditional burial practices [31, 37]. Education messages on fighting the epidemic were communicated through messaging in radio, billboards, etc.

## Conclusions

In conclusion, the 2014 West African Ebola outbreak devastated three West African countries and propagated to seven additional countries on three continents. The public health response and subsequent control of the outbreak was different across Guinea, Liberia and Sierra Leone. The results from both the analysis of country-level data and the qualitative analysis generate hypotheses that inadequate infrastructure at the country level contributed to the delayed control of EVD within the affected countries and contributed to lower survival rates. This Ebola epidemic, as many in the past (such as HIV, West Nile virus, SARS, and cholera in Haiti), shows us that epidemics can have a devastating global impact, and therefore should be considered everyone’s problem. The recent Zika virus epidemic followed many similar patterns highlighting that we all share the responsibility in responding to infectious outbreaks. Many of the country-level factors, particularly the WHO key indicators were associated with controlling the epidemic. International political and public health leaders should assess the infrastructure of countries affected by communicable diseases.

## Methods

We used publicly available data for Guinea, Liberia, and Sierra Leone for the following demographic attributes: 1) population and population density, 2) median age (years), 3) infant mortality rate (deaths/1000 live births), 4) health expenditures (% of GDP), 5) physician density (physicians/1000 population), 6) hospital bed density (beds/1000 population), 7) literacy (age 15+ can read and write), 8) education expenditures (% of GDP), 9) urbanization (% urban), 10) religions (% distribution), and 11) ethnic groups (% distribution). Pearson correlations were calculated and associated scatterplots were generated to assess associations between healthcare indicators for each country (health expenditures, physician density, hospital bed density, and infant mortality rate) and the duration of the EVD outbreak (in days) and the EVD-specific mortality rate.

The outbreak-specific data were obtained from the weekly WHO Ebola Situation Reports during which the key indicator data were uniformly published (March 18, 2015–October 21, 2015). Key indicators included 1) number of confirmed cases, 2) number of confirmed deaths, 3) number and proportion of EVD-positive reported community deaths, 4) number of samples tested and the percent of positive EVD results, 5) percent of new confirmed cases from negative contacts, 6) time between symptom onset and hospitalization (in days), 7) case fatality rate among hospitalized cases, 8) number of newly infected health workers, 9) number of unsafe burials and the reported number of community deaths, 10) number of districts with at least one security incident or other form of refusal to cooperate. Line graphs for each key indicator were generated to assess differences between Guinea, Liberia, and Sierra Leone.

We also summarize the impact of EVD in the seven additional countries with limited EVD transmission. The number of cases, deaths, case fatality rate, case-contacts requiring monitoring, date of first EVD case, and date first declared Ebola-free were summarized for each country. Data were collected using Index Mundi, the WHO Situation Reports and Internet searches, focusing on government sources, to identify relevant Ebola-related information within the scope of outcome measures listed above.

## Abbreviations

CDC: Centers for Disease Control and Prevention
EVD: Ebola virus disease
GDP: gross domestic product
HCW: healthcare worker
U.S.: United States of America
UK: The United Kingdom of Great Britain and Northern Ireland
WHO: World Health Organization
